# The associations between red cell distribution width and plasma proteins in a general population

**DOI:** 10.1186/s12014-021-09319-9

**Published:** 2021-03-30

**Authors:** Jingxue Pan, Yan Borné, Marju Orho-Melander, Jan Nilsson, Olle Melander, Gunnar Engström

**Affiliations:** Department of Clinical Sciences, Lund University, CRC Hus 60 plan 13, Jan Waldenströms gata 35, 20502 Malmö, Sweden

**Keywords:** Population-based, Plasma proteomics, Red cell distribution width, Cardiovascular panel

## Abstract

**Background:**

High red cell distribution width (RDW) has been increasingly recognized as a risk factor for cardiovascular diseases (CVDs), but the underlying mechanisms remain unknown. Our aim was to explore the associations between RDW and plasma proteins implicated in the pathogenesis of CVD using a targeted proteomics panel.

**Methods:**

RDW and 88 plasma proteins were measured in a population-based cohort study (n = 4726), Malmö Diet and Cancer-Cardiovascular Cohort (MDC-CC). A random 2/3 of the cohort was used as discovery sample and remaining 1/3 was used for replication. Multiple linear regression was used to assess the associations between RDW and plasma proteins, with adjustments for age, sex, and other potential confounders. Proteins with Bonferroni-corrected significant associations with RDW in the discovery sub-cohort were validated in the replication cohort.

**Results:**

Thirteen of 88 plasma proteins had significant associations with RDW in the discovery sample, after multivariate adjustments. Eleven of them were also significant in the replication sample, including SIR2-like protein 2 (SIRT2), stem cell factor (SCF, inversely), melusin (ITGB1BP2), growth differentiation factor-15 (GDF-15), matrix metalloproteinase-7 (MMP-7), hepatocyte growth factor (HGF), chitinase-3-like protein 1 (CHI3L1), interleukin-8 (IL-8), CD40 ligand (CD40-L), urokinase plasminogen activator surface receptor (U-PAR) and matrix metalloproteinase-3 (MMP-3).

**Conclusions:**

Several proteins from this targeted proteomics panel were associated with RDW in this cohort. These proteins could potentially be linked to the increased cardiovascular risk in individuals with high RDW.

## Background

Cardiovascular disease (CVD) is one of the leading causes of mortality and morbidity, with substantial impact on the public health worldwide. Beyond the traditional risk factors for CVD, novel circulating blood biomarkers are frequently studied [1, 2], which can be measured with relative ease and are capable of detecting subtle changes in the pathophysiological processes underlying CVDs.

Red cell distribution width (RDW) reflects the heterogeneity of the red blood cells (RBC) volumes, which is often used clinically as a diagnostic tool of patients with anaemia. Since Felker et al. [3] first identified that RDW may be useful for predicting both morbidity and mortality in heart failure (HF) patients, increasing number of studies showed that high RDW is associated with incidence and prevalence of a broad range of CVDs, including atrial fibrillation [4], stroke [5, 6], and myocardial infarction [2, 7]. Though the specific mechanisms between RDW and adverse cardiovascular outcomes have not been sufficiently investigated, it has often been proposed that low-grade inflammation or the actions of pro-inflammatory cytokines could be a common cause of high RDW and CVD [7]. It has been shown that inflammatory cytokines could inhibit the maturation of RBCs [8].

In this population-based study, our primary aim was to explore the potential associations between RDW and a panel of circulating proteins known or suggested to be associated with CVD pathology. The initial analysis was performed in a discovery sub-cohort, consisting of a random 2/3 of the cohort, with adjustment of potential confounding factors. Plasma proteins with significant Bonferroni-corrected relationships with RDW were then confirmed in a validation sub-cohort consisting of the remaining 1/3 of the cohort.

## Methods

### Study population

The Malmö Diet and Cancer (MDC) cohort was established between 1991 and 1996. A total of 30,446 men and women from the city of Malmö were recruited by mail and newspaper advertisement and invited to a health examination at a screening centre. A random 50% of those examined between October 1991 and February 1994 (MDC-Cardiovascular Cohort, MDC-CC, n = 6103), were selected to undergo additional examinations for a cardiovascular sub-study. Of them, 4742 people had information for plasma proteins. In addition, individuals with missing data for RDW (n = 3), smoking (n = 6), high-density lipoprotein (HDL) (n = 1), haemoglobin (HGB) (n = 1), and low-density lipoprotein (LDL) (n = 5) were excluded. After exclusion of missing values, 4726 subjects (1886 men and 2840 women) with average age 57.51 ± 5.96 (mean ± standard deviation, SD) were analysed in our study.

All data were pseudonymized during analytic work with no identity being revealed.

### Baseline examination

Anthropometric measurements were made at baseline using standard procedures. Venous blood samples were drawn at the first visit at the screening centre. HGB, and erythrocyte diameter were analyzed in fresh, heparinized blood, using a fully automated assay (SYSMEX K1000 hematology analyzer; TOA Medical Electronics, Kobe, Japan). RDW was calculated as the width (fL) of the erythrocyte distribution curve at the relative height of 20% above the baseline [9]. Reference values were 36.4–46.3 fL in women and 35.1–43.9 fL in men [10]. The relationships between RDW, cardiovascular risk factors and incidence of cardiovascular disease has been presented in previous papers [4, 5, 7, 11]. Weight and height were measured in light indoor clothing, without shoes. Body mass index (BMI) was calculated as weight/height^2^ (kg/m^2^). Smoking was obtained from the self-administered questionnaire. Smoking was categorized in two categories—smoking (i.e., current or occasional smokers) and non-smoking (never smokers or former smokers). Diabetes was defined as self-reported physician-diagnosed diabetes or current use of diabetes medication or with venous whole blood glucose ≥ 6.1 mmol/L (corresponding to plasma glucose ≥ 7.0 mmol/L).

### Laboratory measurements

HDL and glucose levels were analysed using standard procedures at the Department of Clinical Chemistry, Malmö University Hospital. LDL levels were calculated according to the Friedewald formula. Plasma proteins were measured in fasting EDTA-plasma which had been frozen at − 80 °C after collection at the baseline examination until analysis.

### Proteomics analysis

Ninety-two plasma proteins were analysed using the Olink Proseek Multiplex CVD Panel I 96 × 96 Kit (Olink Bioscience, Uppsala, Sweden), based on the Proximity Extension Assay (PEA) technology with the Fluidigm BioMark HD real-time PCR platform in 54 chip runs. PEA uses matched antibodies labelled with unique oligonucleotides, binding to a targeted protein. This makes probe pairs hybridize and create double-stranded signals, which are amplified and quantified by the PCR platform [12], generating Normalized Protein Expression (NPX) values which corresponds to protein levels. Normalization procedure included a set of internal controls (incubation, extension and detection controls) and a total of 6 external controls for each plate, used to correct for variation between runs and plates (inter-plate controls) and for assessment of detection limits. The protein concentrations are presented as arbitrary units (AU) on a log2 scale. LOD (limit of detection) is defined as 3 × standard deviations (SD) above background based on the negative controls in each run. Protein values below the LOD were replaced with LOD/2. Intra- and inter-assay coefficients of variation for the various proteins, and information regarding the CVD proteomic panel, PEA technology, data normalization and standardization is available in detail on the Olink webpage (http://www.olink.com). Previous studies from this cohort have reported the plasma protein profiles in individuals with high cadmium concentrations and poor self-rated health [13, 14].

Four proteins with less than 75% of subjects having a valid measurement were excluded: Beta-nerve growth factor (Beta-NGF, n = 478); Protein S100-A12 (EN-RAGE, n = 128); Natriuretic peptides B (BNP, n = 696); Interleukin-4 (IL-4, n = 29). In total, 88 cytokines were used for analyses in this study. For analytic purposes, the cohort was divided randomly in an approximate 2:1 ratio (discovery cohort: 2/3 population; validation cohort: 1/3 population).

### Statistical analysis

Data were presented as mean ± SD for continuous variables with normal distribution and percentage for categorical variables. Multiple linear regression was performed to explore the associations between RDW and different proteins (one protein at a time), with RDW as dependent variable and protein as well as other risk factors as independent variables. We used the standardized form of plasma proteins (i.e. the Z-score) to allow for direct comparisons of different biomarkers. Beta coefficients with 95% confidence intervals (CIs) were presented. Model 1 was adjusted for age and sex, model 2 was adjusted for potential confounding factors (i.e. age, sex, BMI, HGB, LDL, HDL, diabetes, smoking). The calculations were performed using IBM SPSS Statistics V.27 (www.spss.com). P value < 5.68 × 10^–4^ (Bonferroni adjustment for 88 tests, 0.05/88) was considered significant in the discovery population (2/3 of subjects). A p value < 0.05 was used as criterion for successful replication in the remaining 1/3 of the population. Since RDW and several plasma proteins are increased by smoking, we also examined the associations with RDW in never smokers as sensitive analysis. This sensitivity analysis was only performed for proteins with significant Bonferroni-adjusted p-values. Pearsons’ correlation test was also performed to examine the relations between each two replicated plasma proteins.

## Results

### Characteristics of subjects

The characteristics of the study population were shown in Table 1. Mean age was 57.5 years, 60% were women, and prevalence of smoking was 22.4% in men and 21.0% in women. The distribution of RDW is illustrated in Additional file 8: Figure S3. The results from the proteomics analysis and a list of the proteins are presented in Additional file 1: Table S1.

**Table 1:** The characteristics of the study population

Variables	Whole cohort (n = 4726)	Discovery cohort (n = 3151)	Replication cohort (n = 1575)
RDW (fL)	40.42 ± 3.28	40.42 ± 3.26	40.40 ± 3.31
BMI (kg/cm^2^)	25.70 ± 3.90	25.71 ± 3.99	25.67 ± 3.72
Haemoglobin (g/L)	141.84 ± 11.44	141.95 ± 11.51	141.62 ± 11.28
HDL (mmol/L)	1.39 ± 0.37	1.38 ± 0.37	1.41 ± 0.38
LDL (mmol/L)	4.17 ± 0.98	4.17 ± 0.98	4.15 ± 0.97
Age (years)	57.51 ± 5.96	57.47 ± 5.99	57.58 ± 5.90
Sex (%)	M (40); F (60)	M(40.4); F (59.6)	M (39); F (61)
Diabetes (%)	M (10.8); F (5.6)	M (10.9); F (5.5)	M (10.4); F (5.6)
Smoking (%)	M (22.4); F (21.0)	M (23.0); F (21.3)	M (21.0); F (20.4)

Values are expressed as mean ± standard deviation (SD) for continuous variables or percentages (%) for categorical variables

### RDW in relation to plasma proteins

#### Discovery sample

**Fig. 1:**
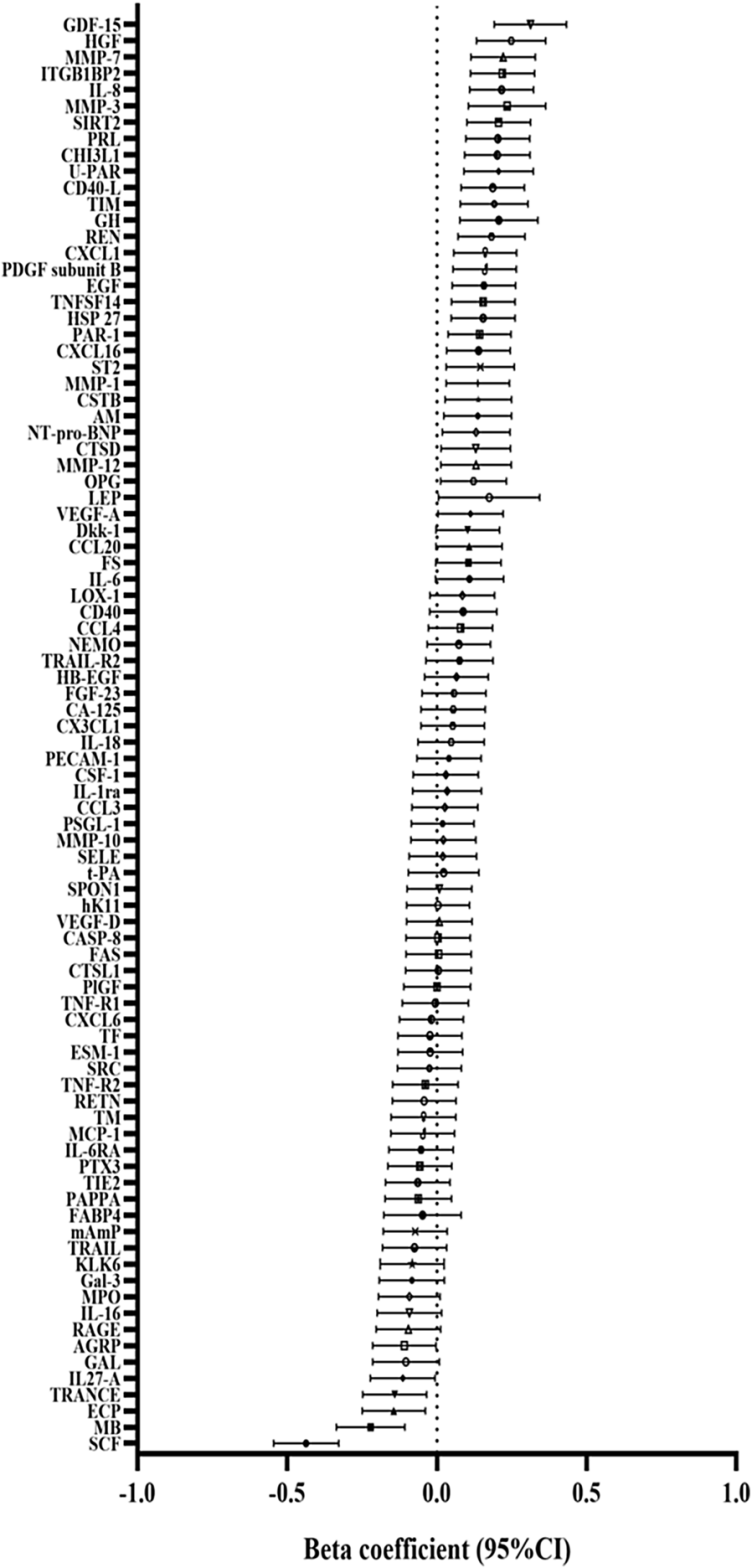
Red cell distribution width in relation to plasma proteins in discovery cohort. The beta coefficient and 95% confidence interval (CI) were obtained from multiple linear regression performed separately for each protein. Adjustments: age, sex, BMI, HGB, LDL, HDL, diabetes, smoking. P < 5.68 × 10^–4^ is significant

Thirty-one of 88 plasma proteins were significantly associated with RDW in the discovery cohort (n = 3151, random 2/3) (p < 5.68 × 10^–4^), with adjustments of age and sex (Additional file 6: Figure S1). Thirteen of 88 plasma proteins showed significant associations with RDW in the discovery population (n = 3151, random 2/3) (p < 5.68 × 10^–4^), after adjustments for age, sex, BMI, HGB, LDL, HDL, diabetes and smoking. The significant proteins were stem cell factor (SCF, inverse association), growth differentiation factor 15 (GDF-15), SIR2-like protein 2 (SIRT2), melusin (ITGB1BP2), matrix metalloproteinase-7 (MMP-7), hepatocyte growth factor (HGF), chitinase-3-like protein 1 (CHI3L1), interleukin-8 (IL-8), CD40 ligand (CD40-L), urokinase plasminogen activator surface receptor (U-PAR), matrix metalloproteinase-3 (MMP-3), prolactin (PRL) and myoglobin (MB). (Fig. 1, Additional file 2: Table S2).

#### Replication sample

**Fig. 2:**
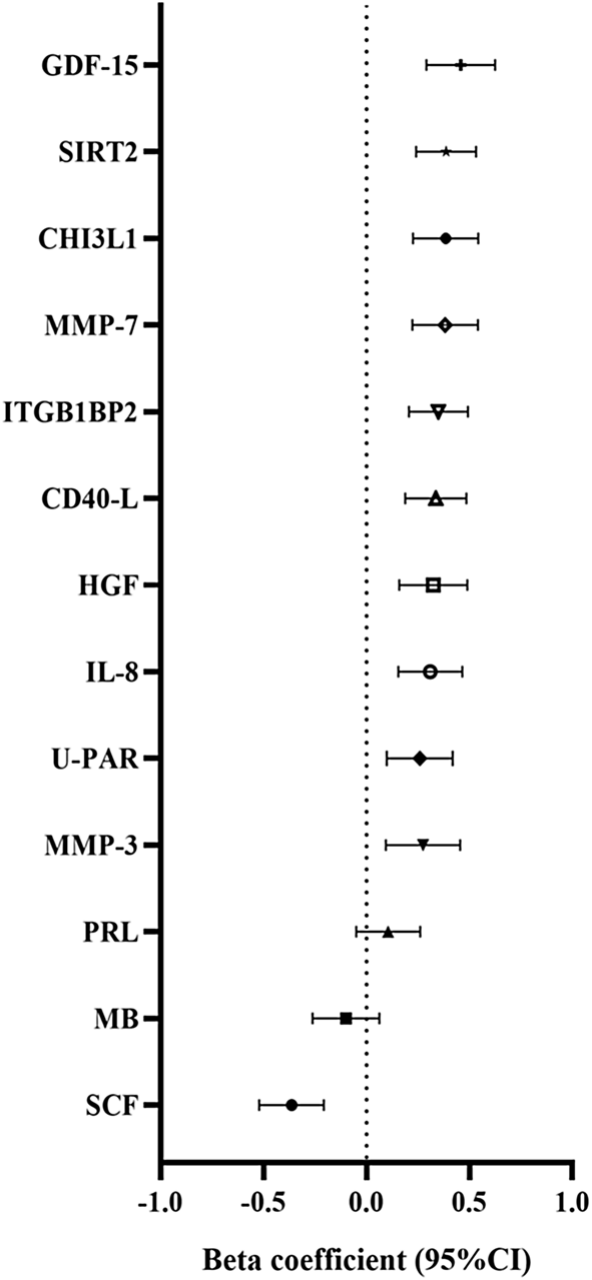
The associations between red cell distribution width and plasma proteins in replication cohort. The beta coefficient and 95% confidence interval (CI) were obtained from multiple linear regression performed separately for each protein. Adjustments: age, sex, BMI, HGB, LDL, HDL, diabetes, smoking. P < 0.05 is significant

Thirteen proteins were assessed for significance in the remaining 1/3 replication sample (n = 1575). Eleven of them were significantly associated with RDW (p < 0.05) (Fig. 2, Additional file 3: Table S3), which included SIRT2, SCF, ITGB1BP2, GDF-15, CHI3L1, CD40-L, MMP-7, IL-8, HGF, U-PAR and MMP-3, respectively. Among them, GDF-15 showed the most significant associations with RDW (beta = 0.46, 95%CI: 0.29–0.63, p = 7.97 × 10^–8^). SCF was inversely associated with RDW (beta = − 0.36, 95% CI: − 0.52— − 0.21), p = 5.59 × 10^–6^). Scatterplots of RDW and four of the significant proteins (GDF-15, SIRT2, CHI3L1, SCF) are presented in Additional file 9: Figure S4.

### Protein–protein correlations

**Fig. 3:**
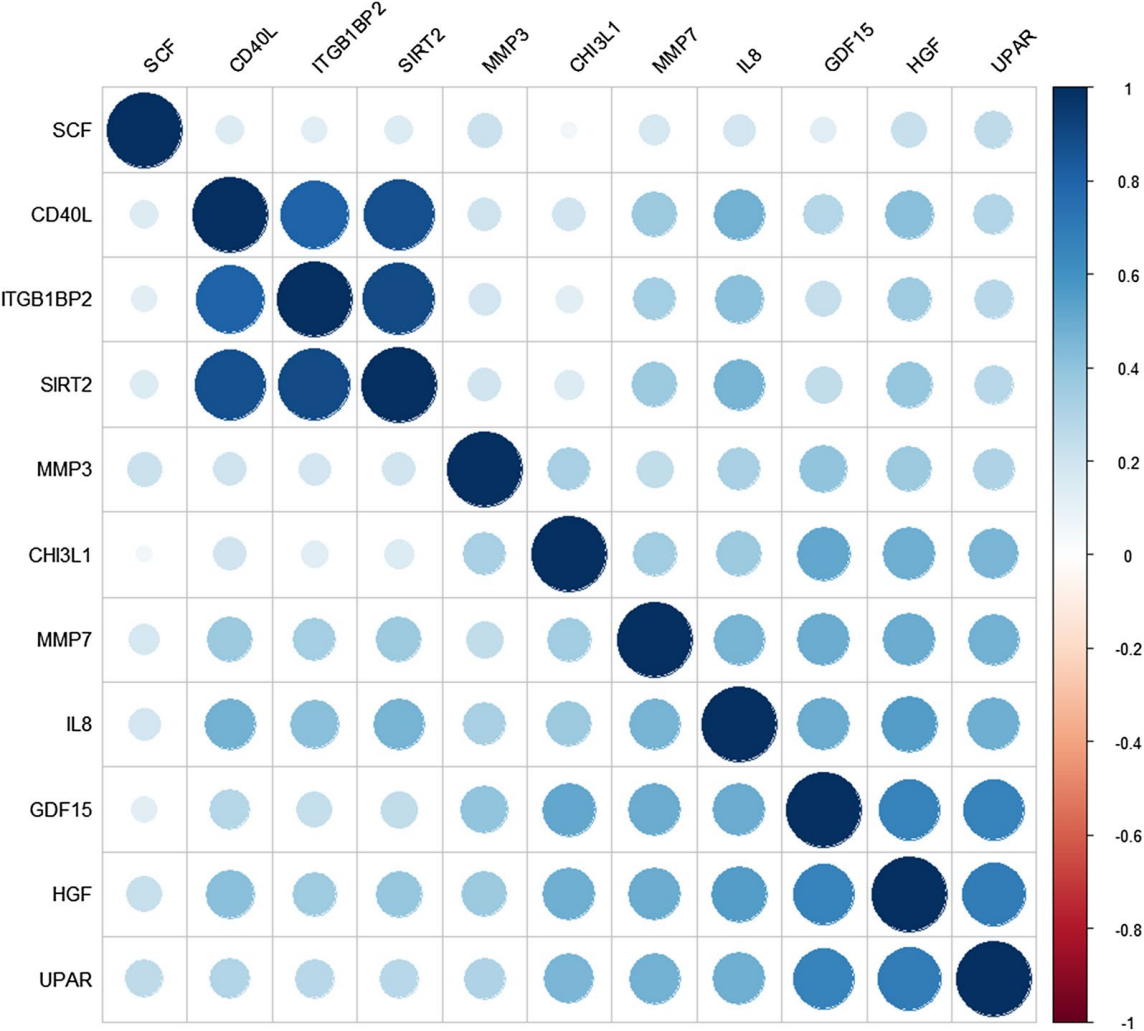
Pair-wise correlations between plasma proteins with significant relationships with RDW. Correlations were assessed between each two proteins using Pearson’s correlation test. Stronger correlation corresponding to darker colour

We analysed the correlations between the plasma proteins with significant relationships with RDW (Fig. 3, Additional file 4: Table S4). We found moderate correlations between most of the proteins and all correlations were statistically significant (p < 0.01).

### Sensitive analysis in never smokers

Eleven of 13 plasma proteins were significantly associated with RDW in never smokers, after multivariate adjustments. Significant proteins in never smokers were ITGB1BP2, SIRT2, PRL, CHI3L1, GDF-15, MMP-7, CD40-L, MMP-3, IL-8, HGF and SCF. The most significant negative associations among them was SCF (beta = − 0.34, 95% CI: − 0.48— − 0.20, p = 2.38 × 10^–6^) (Additional file 5: Table S5, Additional file 7: Figure S2).

## Discussion

Previous studies have linked RDW to a wide range of cardiovascular outcomes and adverse prognosis in patients with CVD [15] and with incidence of CVD in studies from the general population [2, 4–7]. However, the underlying mechanisms by which elevated RDW levels were associated with adverse outcomes remain unclear. The present results show that several proteins with possible associations to CVD are associated with RDW. Eleven proteins (GDF-15, SIRT2, ITGB1BP2, MMP-7, SCF (inversely), CHI3L1, MMP-3, HGF, IL-8, U-PAR, and CD40-L) were significantly associated with RDW after adjustment for possible confounding factors, both in the discovery and replication sample.

High RDW reflects a high heterogeneity of the volumes of RBCs. There could be some principally different reasons for increased RDW values. A high production of large immature erythrocytes, e.g., after major bleeding, is associated with high RDW. Similarly, if the RBCs are heterogeneous already when released into the circulation, this could also cause high RDW. After the reticulocytes have been released, they rapidly develop into smaller erythrocytes, and the volumes of the RBCs then gradually shrink and become smaller over their life span. The average survival time for erythrocytes in the circulation is approximately 120 days, however, it has been shown that there is substantial variations between individuals [16]. Therefore, a high proportion of old and small RBCs could also result in high RDW. Patel et al. [17] proposed that high RDW is a result of delayed elimination of RBCs from the circulation, beyond the average survival time of 120 days. The delayed elimination of erythrocytes could be a physiological response to stress or poor health, with the purpose to save energy and iron to the body [18], and thereby a non-specific marker of disease. This view is supported by the fact that RDW has been positively correlated to haemoglobin A1c (HbA1c), which is influenced by the life-span of the RBCs, but not with plasma glucose [11, 17]. It is not possible to examine the possible reasons for high RDW in this study. However, it is likely that both individuals with a delayed elimination of old erythrocytes and individuals with a high production of large RBCs could be present in this large study from the general population.

It is widely acknowledged that low grade inflammation and chronic disease is associated with anaemia [19] and that several pro-inflammatory cytokines, such as IL-6 and tumor necrosis factor-α (TNF-α) could inhibit erythropoiesis and the actions of erythropoietin [20]. Inflammation includes the production of a wide range of pro-inflammatory cytokines as well as proteins with compensating and regulating effects [21]. It is noteworthy that several of the proteins associated with RDW in this study can be regarded as markers of inflammation (e.g. GDF-15, CD40-L, IL-8, MMP-3, MMP-7, U-PAR and CHI3L1), and it is possible that they were related to erythropoiesis, either directly, or through other proteins in the inflammatory cascade. It is also noteworthy that many of these plasma proteins have been associated with increased risk of CVD [22–28].

GDF-15 is a protein with many functions, which has been reported to be elevated in various anaemic conditions [29]. GDF-15 has a role in regulation of iron homeostasis [30] and it has been shown that GDF-15 expression is regulated by hypoxia [31] and iron depletion [32]. This could potentially explain its relationship with RDW in our study. It is also noteworthy that GDF-15 has been repeatedly associated with all-cause mortality [33] and CVD [22].

HGF has multiple pleiotropic effects on cardiovascular system [34, 35] and is involved in activation of hematopoietic progenitor cells. It has been shown that HGF is produced by human bone marrow stroma cells and promotes proliferation, adhesion and survival of human haematopoietic progenitor cells [36–38]. It was recently shown that HGF is associated with progression of atherosclerosis, which perhaps could reflect a compensatory and repairing function of HGF in atherosclerosis [35].

By contrast, there was negative association between RDW and SCF. SCF is a major stimulator of erythropoiesis and works synergistically together with erythropoietin [39]. It could be speculated that low levels of SCF might be an indicator of reduced renewal of erythrocytes in the circulation and an increased population of older and smaller RBCs, which in turn, could explain the negative correlation between RDW and SCF [40, 41]. It is noteworthy that low SCF was associated with increased risk of CVD in a recent study from the MDC-cohort [42].

### Strengths and limitations

We used a well-defined population-based cohort with extensive information about plasma proteins as well as potential confounding factors. The sample size was big enough to allow analysis of a random discovery and replication sample. The PEA technology is known to be very specific, however, the concentrations are given as arbitrary units and not in International System of Units (SI units), which is a limitation. The present study included 88 plasma proteins which could be linked to CVD. However, we have no information about protein biomarkers for other diseases. Whether RDW is associated with biomarkers for other diseases should be examined in future studies. Another limitation is that the analysis was performed in a cross-sectional design and we can only speculate about causality.

## Conclusions

In our prospective cohort study, eleven of 88 plasma proteins showed significant associations with RDW which were replicated successfully in validation sub-cohort. These proteins could be related to the increased cardiovascular risk in individuals with high RDW.

## Supplementary Information

**Additional file 1: Table S1.** Circulating protein levels in the whole population.

**Additional file 2: Table S2.** Red cell distribution width in relation to plasma proteins in discovery sample.

**Additional file 3: Table S3.** The associations between red cell distribution width and plasma proteins in replication sample.

**Additional file 4: Table S4.** Pair-wise correlations between plasma proteins with significant relationships with RDW.

**Additional file 5: Table S5.** The associations between red cell distribution width and plasma proteins among never smokers.

**Additional file 6: Figure S1.** Red cell distribution width in relation to plasma proteins in discovery cohort.

**Additional file 7: Figure S2.** The associations between red cell distribution width and plasma proteinsamong never smokers.

**Additional file 8: Figure S3.** Distribution plot for RDW.

**Additional file 9: Figure S4.** The scatter plots for RDW and four validated proteins – (A) GDF-15, (B) SIRT2, (C) CHI3L1 and (D) SCF.

## Data Availability

The dataset in the current study is available for research purposes after application to the MDCS steering committee, Lund University (email to anders.dahlin@med.lu.se).

## Text

AU: Arbitrary units
BMI: Body mass index
CVDs: Cardiovascular diseases
CI: Confidence interval
HbA1c: Haemoglobin A1c
HGB: Haemoglobin
HDL: High-density lipoprotein
HF: Heart failure
LDL: Low-density lipoprotein
LOD: Limit of detection
MDC-CC: Malmö Diet and Cancer—Cardiovascular Cohort
NPX: Normalized Protein Expression
PEA: Proximity Extension Assay technology
RDW: Red cell distribution width
RBC: Red blood cell
SD: Standard deviation
SI units: International System of Units
TNF-α: Tumor necrosis factor-α

## Plasma proteins

AGRP: Agouti-related protein
AM: Adrenomedullin
Beta-NGF: Beta-nerve growth factor
BNP: Natriuretic peptides B
CA-125: Ovarian cancer-related tumor marker CA 125
CASP-8: Caspase-8
CCL20: C–C motif chemokine 20
CCL3: C–C motif chemokine 3
CCL4: C–C motif chemokine 4
CD40: CD40 ligand receptor
CD40-L: CD40 ligand
CHI3L1: Chitinase-3-like protein 1
CSF-1: Macrophage colony-stimulating factor 1
CSTB: Cystatin-B
CTSD: Cathepsin D
CTSL1: Cathepsin L1
CX3CL1: Fractalkine
CXCL1: C–X–C motif chemokine 1
CXCL16: C–X–C motif chemokine 16
CXCL6: C–X–C motif chemokine 6
DKK-1: Dickkopf-related protein 1
ECP: Eosinophil cationic protein
EGF: Epidermal growth factor
EN-RAGE: Protein S100-A12
ESM-1: Endothelial cell-specific molecule 1
FABP4: Fatty acid-binding protein, adipocyte
FAS: Tumor necrosis factor receptor superfamily member 6
FGF-23: Fibroblast growth factor 23
FS: Follistatin
GAL: Galanin peptides
Gal-3: Galectin-3
GDF-15: Growth/differentiation factor 15
GH: Growth hormone
HB-EGF: Heparin-binding EGF-like growth factor
HGF: Hepatocyte growth factor
hK11: Kallikrein-11
HSP27: Heat shock 27 kDa protein
IL-16: Interleukin-16
IL-18: Interleukin-18
IL-1RA: Interleukin-1 receptor antagonist protein
IL-4: Interleukin-4
IL-6: Interleukin-6
IL-6RA: Interleukin-6 receptor subunit alpha
IL-8: Interleukin-8
IL27-A: Interleukin-27 subunit alpha
ITGB1BP2: Melusin
KLK6: Kallikrein-6
LEP: Leptin
LOX-1: Lectin-like oxidized LDL receptor 1
mAmp: Membrane-bound aminopeptidase P
MB: Myoglobin
MCP-1: Monocyte chemotactic protein 1
MMP-1: Matrix metalloproteinase-1
MMP-10: Matrix metalloproteinase-10
MMP-12: Matrix metalloproteinase-12
MMP-3: Matrix metalloproteinase-3
MMP-7: Matrix metalloproteinase-7
MPO: Myeloperoxidase
NEMO: NF-kappa-B essential modulator
NT-pro-BNP: N-terminal pro-B-type natriuretic peptide
OPG: Osteoprotegerin
PAPPA: Pappalysin-1
PAR-1: Proteinase-activated receptor 1
PDGF subunit B: Platelet-derived growth factor subunit B
PECAM-1: Platelet endothelial cell adhesion molecule
PlGF: Placenta growth factor
PRL: Prolactin
PSGL-1: P-selectin glycoprotein ligand 1
PTX3: Pentraxin-related protein PTX3
RAGE: Receptor for advanced glycosylation end products
REN: Renin
RETN: Resistin
SCF: Stem cell factor
SELE: E-selectin
SIRT2: SIR2-like protein 2
SPON1: Spondin-1
SRC: Proto-oncogene tyrosine-protein kinase Src
ST2: ST2 protein
t-PA: Tissue-type plasminogen activator
TF: Tissue factor
TIE2: Angiopoietin-1 receptor
TIM: T cell immunoglobin domain and mucin domain protein 1 (Kidney injury molecule 1)
TM: Thrombomodulin
TNF-R1: Tumor necrosis factor receptor 1
TNF-R2: Tumor necrosis factor receptor 2
TNFSF14: Tumor necrosis factor ligand superfamily member 14
TRAIL: TNF-related apoptosis-inducing ligand
TRAIL-R2: TNF-related apoptosis-inducing ligand receptor 2
TRANCE: TNF-related activation-induced cytokine
U-PAR: Urokinase plasminogen activator surface receptor
VEGF-A: Vascular endothelial growth factor A
VEGF-D: Vascular endothelial growth factor D

## References

[1] Pilling LC, Atkins JL, Kuchel GA, Ferrucci L, Melzer D (2018). Red cell distribution width and common disease onsets in 240,477 healthy volunteers followed for up to 9 years. PLoS ONE.

[2] Skjelbakken T, Lappegård J, Ellingsen TS, Barrett-Connor E, Brox J, Løchen ML, Njølstad I, Wilsgaard T, Mathiesen EB, Brækkan SK, Hansen JB (2014). Red cell distribution width is associated with incident myocardial infarction in a general population: the Tromsø Study. J Am Heart Assoc.

[3] Felker GM, Allen LA, Pocock SJ, Shaw LK, McMurray JJ, Pfeffer MA, Swedberg K, Wang D, Yusuf S, Michelson EL, Granger CB (2007). Red cell distribution width as a novel prognostic marker in heart failure: data from the CHARM Program and the Duke Databank. J Am Coll Cardiol.

[4] Adamsson Eryd S, Borné Y, Melander O, Persson M, Smith JG, Hedblad B, Engström G (2014). Red blood cell distribution width is associated with incidence of atrial fibrillation. J Intern Med.

[5] Söderholm M, Borné Y, Hedblad B, Persson M, Engström G (2015). Red cell distribution width in relation to incidence of stroke and carotid atherosclerosis: a population-based cohort study. PLoS ONE.

[6] Lappegård J, Ellingsen TS, Skjelbakken T, Mathiesen EB, Njølstad I, Wilsgaard T, Brox J, Brækkan SK, Hansen JB (2016). Red cell distribution width is associated with future risk of incident stroke. The Tromsø Study Thromb Haemost.

[7] Borné Y, Smith JG, Melander O, Engström G (2014). Red cell distribution width in relation to incidence of coronary events and case fatality rates: a population-based cohort study. Heart.

[8] Pierce CN, Larson DF (2005). Inflammatory cytokine inhibition of erythropoiesis in patients implanted with a mechanical circulatory assist device. Perfusion.

[9] Constantino BT (2013). Red Cell Distribution Width, Revisited. Laboratory Medicine.

[10] RDW-SD and RDW-CV: using this information practically. Sysmex Xtra Online March 2011. https://www.sysmex.de/fileadmin/media/f101/Xtra/Themenblaetter/11.2.01_RDW-SD_und_RDW-CV_RZ_Web.pdf. Accessed 9 Febr 2021.

[11] Engström G, Smith JG, Persson M, Nilsson PM, Melander O, Hedblad B (2014). Red cell distribution width, haemoglobin A1c and incidence of diabetes mellitus. J Intern Med.

[12] Assarsson E, Lundberg M, Holmquist G, Björkesten J, Bucht Thorsen S, Ekman D, Eriksson A, Rennel Dickens E, Ohlsson S, Edfeldt G (2014). Homogenous 96-Plex PEA immunoassay exhibiting high sensitivity, specificity, and excellent scalability. PLoS ONE.

[13] Bao X, Borné Y, Yin S, Niu K, Orho-Melander M, Nilsson J, Melander O, Engström G (2019). The associations of self-rated health with cardiovascular risk proteins: a proteomics approach. Clin Proteomics.

[14] Borné Y, Fagerberg B, Sallsten G, Hedblad B, Persson M, Melander O, Nilsson J, Orho-Melander M, Barregard L, Engström G (2019). Biomarkers of blood cadmium and incidence of cardiovascular events in non-smokers: results from a population-based proteomics study. Clin Proteomics.

[15] Pascual-Figal DA, Bonaque JC, Redondo B, Caro C, Manzano-Fernandez S, Sanchez-Mas J, Garrido IP, Valdes M (2009). Red blood cell distribution width predicts long-term outcome regardless of anaemia status in acute heart failure patients. Eur J Heart Fail.

[16] Furne JK, Springfield JR, Ho SB, Levitt MD (2003). Simplification of the end-alveolar carbon monoxide technique to assess erythrocyte survival. J Lab Clin Med.

[17] Patel HH, Patel HR, Higgins JM (2015). Modulation of red blood cell population dynamics is a fundamental homeostatic response to disease. Am J Hematol.

[18] Weiss G, Goodnough LT (2005). Anemia of chronic disease. N Engl J Med.

[19] Fraenkel PG (2017). Anemia of inflammation: a review. Med Clin North Am.

[20] Smrzova J, Balla J, Barany P (2005). Inflammation and resistance to erythropoiesis-stimulating agents–what do we know and what needs to be clarified?. Nephrol Dial Transplant.

[21] Gabay C, Kushner I (1999). Acute-phase proteins and other systemic responses to inflammation. N Engl J Med.

[22] Andersson C, Enserro D, Sullivan L, Wang TJ, Januzzi JL, Benjamin EJ, Vita JA, Hamburg NM, Larson MG, Mitchell GF, Vasan RS (2016). Relations of circulating GDF-15, soluble ST2, and troponin-I concentrations with vascular function in the community: The Framingham Heart Study. Atherosclerosis.

[23] Michel NA, Zirlik A, Wolf D (2017). CD40L and Its Receptors in Atherothrombosis-An Update. Front Cardiovasc Med.

[24] Djuric T, Zivkovic M, Radak D, Jekic D, Radak S, Stojkovic L, Raicevic R, Stankovic A, Alavantic D (2008). Association of MMP-3 5A/6A gene polymorphism with susceptibility to carotid atherosclerosis. Clin Biochem.

[25] Ridker PM, Chasman DI, Rose L, Loscalzo J, Elias JA (2014). Plasma levels of the proinflammatory chitin-binding glycoprotein YKL-40, variation in the chitinase 3-like 1 gene (CHI3L1), and incident cardiovascular events. J Am Heart Assoc.

[26] Nymo SH, Hulthe J, Ueland T, McMurray J, Wikstrand J, Askevold ET, Yndestad A, Gullestad L, Aukrust P (2014). Inflammatory cytokines in chronic heart failure: interleukin-8 is associated with adverse outcome. Results from CORONA. Eur J Heart Fail.

[27] Tuomainen AM, Kormi I, Havulinna AS, Tervahartiala T, Salomaa V, Sorsa T, Pussinen PJ (2014). Serum tissue-degrading proteinases and incident cardiovascular disease events. Eur J Prev Cardiol.

[28] Persson M, Ostling G, Smith G, Hamrefors V, Melander O, Hedblad B, Engstrom G (2014). Soluble urokinase plasminogen activator receptor: a risk factor for carotid plaque, stroke, and coronary artery disease. Stroke.

[29] Waalen J, von Lohneysen K, Lee P, Xu X, Friedman JS (2011). Erythropoietin, GDF15, IL6, hepcidin and testosterone levels in a large cohort of elderly individuals with anaemia of known and unknown cause. Eur J Haematol.

[30] Tanno T, Bhanu NV, Oneal PA, Goh SH, Staker P, Lee YT, Moroney JW, Reed CH, Luban NL, Wang RH (2007). High levels of GDF15 in thalassemia suppress expression of the iron regulatory protein hepcidin. Nat Med.

[31] Albertoni M, Shaw PH, Nozaki M, Godard S, Tenan M, Hamou MF, Fairlie DW, Breit SN, Paralkar VM, de Tribolet N (2002). Anoxia induces macrophage inhibitory cytokine-1 (MIC-1) in glioblastoma cells independently of p53 and HIF-1. Oncogene.

[32] Lakhal S, Talbot NP, Crosby A, Stoepker C, Townsend AR, Robbins PA, Pugh CW, Ratcliffe PJ, Mole DR (2009). Regulation of growth differentiation factor 15 expression by intracellular iron. Blood.

[33] Bao X, Borné Y, Xu B, Orho-Melander M, Nilsson J, Melander O, Engström G: Growth differentiation factor-15 is a biomarker for all-cause mortality but less evident for cardiovascular outcomes: a prospective study: Abbreviated title: GDF-15, all-cause mortality and CVD. *Am Heart J* 2021. 10.1016/j.ahj.2020.12.020 33421373

[34] Gallo S, Sala V, Gatti S, Crepaldi T (2015). Cellular and molecular mechanisms of HGF/Met in the cardiovascular system. Clin Sci (Lond).

[35] Bell EJ, Decker PA, Tsai MY, Pankow JS, Hanson NQ, Wassel CL, Larson NB, Cohoon KP, Budoff MJ, Polak JF (2018). Hepatocyte growth factor is associated with progression of atherosclerosis: The Multi-Ethnic Study of Atherosclerosis (MESA). Atherosclerosis.

[36] Weimar IS, Miranda N, Muller EJ, Hekman A, Kerst JM, de Gast GC, Gerritsen WR (1998). Hepatocyte growth factor/scatter factor (HGF/SF) is produced by human bone marrow stromal cells and promotes proliferation, adhesion and survival of human hematopoietic progenitor cells (CD34+). Exp Hematol.

[37] Iguchi T, Sogo S, Hisha H, Taketani S, Adachi Y, Miyazaki R, Ogata H, Masuda S, Sasaki R, Ito M (1999). HGF activates signal transduction from EPO receptor on human cord blood CD34+/CD45+ cells. Stem Cells.

[38] Puddighinu G, D'Amario D, Foglio E, Manchi M, Siracusano A, Pontemezzo E, Cordella M, Facchiano F, Pellegrini L, Mangoni A (2018). Molecular mechanisms of cardioprotective effects mediated by transplanted cardiac ckit(+) cells through the activation of an inflammatory hypoxia-dependent reparative response. Oncotarget.

[39] Sui X, Krantz SB, You M, Zhao Z (1998). Synergistic activation of MAP kinase (ERK1/2) by erythropoietin and stem cell factor is essential for expanded erythropoiesis. Blood.

[40] Sui X, Krantz SB, Zhao ZJ (2000). Stem cell factor and erythropoietin inhibit apoptosis of human erythroid progenitor cells through different signalling pathways. Br J Haematol.

[41] Arcasoy MO, Jiang X (2005). Co-operative signalling mechanisms required for erythroid precursor expansion in response to erythropoietin and stem cell factor. Br J Haematol.

[42] Björkbacka H, Yao Mattisson I, Wigren M, Melander O, Fredrikson GN, Bengtsson E, Gonçalves I, Almgren P, Lagerstedt JO, Orho-Melander M (2017). Plasma stem cell factor levels are associated with risk of cardiovascular disease and death. J Intern Med.

