# Supplementary material for: The associations between red cell distribution width and plasma proteins in a general population
Source: Clin Proteomics. 2021 Mar 30;18:12. doi: 10.1186/s12014-021-09319-9 (PMC8008679; doi:10.1186/s12014-021-09319-9)
Supplement: Supplementary file 1 — Additional file 1: Table S1. Circulating protein levels in the whole population. [file 12014_2021_9319_MOESM1_ESM.pdf]

Table S1 Circulating protein levels in the whole population

Proteins		Range (low-high)	Mean ±SD
AGRP	Agouti-related protein	5.1(2.3-7.4)	4.2±0.4
AM	Adrenomedullin	5.2(2.9-8.1)	6.1±0.5
CA-125	Ovarian cancer-related tumor marker CA 125	5.9(1.3-7.3)	3.7±1.0
CASP-8	Caspase-8	4.9(0.1-5.0)	1.5±0.7
CCL20	C-C motif chemokine 20	9.7(2.1-11.8)	5.5±1.0
CCL3	C-C motif chemokine 3	7.6(0.1-7.7)	1.1±0.5
CCL4	C-C motif chemokine 4	7.4(4.7-12.1)	7.2±0.7
CD40	CD40 ligand receptor	5.0(7.5-12.5)	9.4±0.6
CD40-L	CD40 ligand	8.3(3.4-11.7)	7.8±1.0
CHI3L1	Chitinase-3-like protein 1	8.5(1.6-10.1)	5.3±0.9
CSF-1	Macrophage colony-stimulating factor 1	4.1(6.1-10.2)	8.5±0.3
CSTB	Cystatin-B	8.0(1.0-8.9)	4.7±0.6
CTSD	Cathepsin D	4.9(4.0-8.9)	6.8±0.5
CTSL1	Cathepsin L1	4.1(3.5-7.5)	5.5±0.4
CX3CL1	Fractalkine	4.1(3.1-7.2)	5.0±0.5
CXCL1	C-X-C motif chemokine 1	6.2(4.2-10.4)	7.4±0.7
CXCL16	C-X-C motif chemokine 16	3.9(0.9-4.8)	2.8±0.4
CXCL6	C-X-C motif chemokine 6	6.6(3.4-10.0)	6.5±0.9
DKK-1	Dickkopf-related protein 1	5.4(3.2-8.7)	5.7±0.6
ECP	Eosinophil cationic protein	6.7(0.8-7.5)	5.0±0.8
EGF	Epidermal growth factor	8.2(2.4-10.6)	7.1±0.9
ESM-1	Endothelial cell-specific molecule 1	4.4(0.9-5.3)	2.9±0.5
FABP4	Fatty acid-binding protein, adipocyte	4.5(0.7-5.2)	2.4±0.6
FAS	Tumor necrosis factor receptor superfamily member 6	6.3(4.9-11.2)	7.3±0.5
FGF-23	Fibroblast growth factor 23	7.1(-0.2-6.9)	1.9±0.5
FS	Follistatin	5.3(2.2-7.5)	4.8±0.5
GAL	Galanin peptides	6.3(1.4-7.6)	5.0±0.8
Gal-3	Galectin-3	4.1(3.0-7.1)	5.1±0.5
GDF-15	Growth/differentiation factor 15	6.2(6.1-12.3)	8.8±0.6
GH	Growth hormone	9.5(2.8-12.3)	8.3±2.3
HB-EGF	Heparin-binding EGF-like growth factor	3.6(2.9-6.5)	4.6±0.4
HGF	Hepatocyte growth factor	6.2(3.9-10.1)	6.2±0.5
hK11	Kallikrein-11	7.7(1.2-8.9)	4.9±0.5
HSP27	Heat shock 27 kDa protein	6.1(0.5-6.7)	4.7±0.9
IL-16	Interleukin-16	5.2(1.3-6.4)	3.8±0.5
IL-18	Interleukin-18	6.0(6.7-12.7)	9.9±0.6
IL-1RA	Interleukin-1 receptor antagonist protein	7.4(0.2-7.6)	3.7±1.5
IL-6	Interleukin-6	10.2(1.8-12.0)	4.4±1.0
IL-6RA	Interleukin-6 receptor subunit alpha	3.7(6.6-10.3)	8.8±0.5
IL-8	Interleukin-8	9.9(1.3-11.2)	4.7±0.6
IL27-A	Interleukin-27 subunit alpha	3.1(0.2-3.3)	1.8±0.4
ITGB1BP2	Melusin	7.4(1.3-8.7)	4.2±1.0
KLK6	Kallikrein-6	4.9(3.8-8.8)	6.3±0.5

LEP	Leptin	5.4(0.1-5.4)	2.7±1.1
LOX-1	Lectin-like oxidized LDL receptor 1	5.5(1.9-7.4)	4.1±0.6
mAmp	Membrane-bound aminopeptidase P	4.6(0.8-5.3)	2.3±1.1
MB	Myoglobin	5.0(3.1-8.1)	5.4±0.6
MCP-1	Monocyte chemotactic protein 1	5.9(0.5-6.5)	2.5±0.5
MMP-1	Matrix metalloproteinase-1	7.7(-0.04-7.7)	2.9±1.0
MMP-10	Matrix metalloproteinase-10	7.0(3.3-10.3)	6.0±0.7
MMP-12	Matrix metalloproteinase-12	6.3(3.6-9.8)	6.6±0.7
MMP-3	Matrix metalloproteinase-3	5.3(-0.9-4.4)	0.2±0.6
MMP-7	Matrix metalloproteinase-7	7.2(1.2-8.4)	5.5±0.7
MPO	Myeloperoxidase	4.2(0.6-4.8)	3.5±0.4
NEMO	NF-kappa-B essential modulator	7.3(1.0-8.3)	5.0±0.9
NT-pro-BNP	N-terminal pro-B-type natriuretic peptide	6.2(1.1-7.3)	3.2±1.3
OPG	Osteoprotegerin	4.4(6.9-11.3)	9.3±0.4
PAPPA	Pappalysin-1	4.8(0.4-5.2)	1.8±0.6
PAR-1	Proteinase-activated receptor 1	3.7(5.9-9.6)	8.3±0.4
PDGF subunit B	Platelet-derived growth factor subunit B	5.8(5.4-11.2)	9.1±0.7
PECAM-1	Platelet endothelial cell adhesion molecule	4.0(4.2-8.3)	6.3±0.5
PlGF	Placenta growth factor	4.4(4.6-9.1)	7.0±0.4
PRL	Prolactin	6.8(0.6-7.5)	4.2±0.8
PSGL-1	P-selectin glycoprotein ligand 1	1.4(0.3-1.7)	0.6±0.3
PTX3	Pentraxin-related protein PTX3	3.7(0.2-3.9)	1.2±0.5
RAGE	Receptor for advanced glycosylation end products	4.7(1.6-6.3)	4.3±0.4
REN	Renin	6.1(3.5-9.7)	6.5±0.8
RETN	Resistin	6.7(3.4-10.1)	6.0±0.6
SCF	Stem cell factor	4.1(4.1-8.1)	6.8±0.5
SELE	E-selectin	8.4(2.6-11.0)	5.0±0.7
SIRT2	SIR2-like protein 2	7.9(0.5-8.4)	4.7±1.0
SPON1	Spondin-1	4.7(1.9-6.6)	4.3±0.4
SRC	Proto-oncogene tyrosine-protein kinase Src	5.9(4.0-9.9)	7.6±0.3
ST2	ST2 protein	5.2(0.3-5.5)	2.7±0.6
t-PA	Tissue-type plasminogen activator	6.4(2.4-8.8)	5.3±0.8
TF	Tissue factor	4.5(3.5-8.0)	5.6±0.4
TIE2	Angiopoietin-1 receptor	3.7(4.1-7.8)	6.1±0.4
TIM	T cell immunoglobin domain and mucin domain protein 1 (Kidney injury molecule 1)	9.4(1.1-10.4)	5.1±0.8
TM	Thrombomodulin	4.2(6.7-10.9)	9.3±0.4
TNF-R1	Tumor necrosis factor receptor 1	4.4(9.7-14.1)	12.2±0.4
TNF-R2	Tumor necrosis factor receptor 2	4.7(2.3-7.0)	4.9±0.5
TNFSF14	Tumor necrosis factor ligand superfamily member 14	5.2(0.1-5.3)	2.3±0.6
TRAIL	TNF-related apoptosis-inducing ligand	4.8(5.9-10.7)	8.3±0.4
TRAIL-R2	TNF-related apoptosis-inducing ligand receptor 2	9.2(-0.3-8.9)	1.3±0.4
TRANCE	TNF-related activation-induced cytokine	5.0(1.2-6.2)	3.9±0.7
U-PAR	Urokinase plasminogen activator surface receptor	3.7(7.5-11.3)	9.5±0.4
VEGF-A	Vascular endothelial growth factor A	5.1(7.5-12.6)	10.0±0.5
VEGF-D	Vascular endothelial growth factor D	7.8(0.7-8.5)	6.7±0.5

Proteins excluded due to few measurements above lower limit of detection:			
Beta-NGF	Beta-nerve growth factor		
BNP	Natriuretic peptides B		
EN-RAGE	Protein S100-A12		
IL-4	Interleukin-4		

Units: arbitrary units (AU), log2 scale. Range (low to high) and mean (±standard deviation, SD) values of each protein were presented.
