# Supplementary material for: The associations between red cell distribution width and plasma proteins in a general population
Source: Clin Proteomics. 2021 Mar 30;18:12. doi: 10.1186/s12014-021-09319-9 (PMC8008679; doi:10.1186/s12014-021-09319-9)
Supplement: Supplementary file 2 — Additional file 2: Table S2. Red cell distribution width in relation to plasma proteins in discovery sample. [file 12014_2021_9319_MOESM2_ESM.pdf]

Table S2 Red cell distribution width in relation to plasma proteins in discovery sample

	Plasma proteins	Beta coefficient	95% Confidence Interval		P value
			Lower bound	Upper bound	
1	SCF*	-0.44	-0.55	-0.33	4.50×10^{-15}
2	GDF-15*	0.31	0.19	0.43	4.00×10^{-7}
3	HGF*	0.25	0.13	0.36	2.63×10^{-5}
4	MMP-7*	0.22	0.11	0.33	5.45×10^{-5}
5	ITGB1BP2*	0.22	0.11	0.33	6.24×10^{-5}
6	IL-8*	0.22	0.11	0.32	6.92×10^{-5}
7	SIRT2*	0.21	0.10	0.31	1.42×10^{-4}
8	MB*	-0.22	-0.34	-0.11	1.44×10^{-4}
9	PRL*	0.20	0.10	0.31	1.84×10^{-4}
10	CHI3L1*	0.20	0.09	0.31	2.93×10^{-4}
11	MMP-3*	0.23	0.11	0.36	3.85×10^{-4}
12	U-PAR*	0.21	0.09	0.32	4.87×10^{-4}
13	CD40-L*	0.19	0.08	0.29	5.43×10^{-4}
14	TIM	0.19	0.08	0.30	8.89×10^{-4}
15	REN	0.18	0.07	0.29	1.36×10^{-3}
16	GH	0.21	0.08	0.34	1.83×10^{-3}
17	CXCL1	0.16	0.06	0.27	2.61×10^{-3}
18	PDGF subunit B	0.16	0.05	0.27	3.09×10^{-3}
19	EGF	0.16	0.05	0.26	3.59×10^{-3}
20	TNFSF14	0.15	0.05	0.26	4.25×10^{-3}
21	HSP 27	0.15	0.05	0.26	4.49×10^{-3}
22	ECP	-0.14	-0.25	-0.04	7.50×10^{-3}
23	PAR-1	0.14	0.04	0.25	7.64×10^{-3}
24	TRANCE	-0.14	-0.25	-0.03	9.67×10^{-3}
25	CXCL16	0.14	0.03	0.24	1.04×10^{-2}
26	MMP-1	0.14	0.03	0.24	1.14×10^{-2}
27	ST2	0.14	0.03	0.26	1.26×10^{-2}
28	CSTB	0.14	0.03	0.25	1.42×10^{-2}
29	AM	0.14	0.02	0.25	1.80×10^{-2}
30	NT-pro-BNP	0.13	0.02	0.24	2.27×10^{-2}
31	CTSD	0.13	0.01	0.25	2.75×10^{-2}
32	MMP-12	0.13	0.01	0.25	2.94×10^{-2}
33	OPG	0.12	0.01	0.23	2.98×10^{-2}
34	IL27-A	-0.11	-0.22	-0.006	3.86×10^{-2}
35	VEGF-A	0.11	0.004	0.22	4.26×10^{-2}
36	LEP	0.17	0.006	0.34	4.27×10^{-2}
37	AGRP	-0.11	-0.21	-0.003	4.34×10^{-2}
38	CCL20	0.11	-0.003	0.22	5.68×10^{-2}
39	Dkk-1	0.10	-0.003	0.21	0.057
40	FS	0.11	-0.004	0.21	0.059
41	IL-6	0.11	-0.005	0.22	0.060

42	GAL	-0.10	-0.21	0.008	0.069
43	MPO	-0.09	-0.19	0.01	0.079
44	RAGE	-0.09	-0.20	0.01	0.083
45	IL-16	-0.09	-0.20	0.02	0.093
46	CD40	0.09	-0.02	0.20	0.124
47	LOX-1	0.08	-0.02	0.19	0.125
48	KLK6	-0.08	-0.19	0.02	0.130
49	Gal-3	-0.08	-0.19	0.02	0.131
50	CCL4	0.08	-0.03	0.19	0.148
51	TRAIL	-0.07	-0.18	0.03	0.171
52	NEMO	0.07	-0.03	0.18	0.174
53	mAmP	-0.07	-0.18	0.03	0.183
54	TRAIL-R2	0.08	-0.04	0.19	0.186
55	HB-EGF	0.07	-0.04	0.17	0.227
56	TIE2	-0.06	-0.17	0.04	0.245
57	PAPPA	-0.06	-0.17	0.05	0.270
58	FGF-23	0.06	-0.05	0.16	0.291
59	PTX3	-0.06	-0.16	0.05	0.296
60	CA-125	0.05	-0.05	0.16	0.319
61	CX3CL1	0.05	-0.05	0.16	0.326
62	IL-6RA	-0.05	-0.16	0.05	0.332
63	MCP-1	-0.05	-0.15	0.06	0.382
64	IL-18	0.05	-0.06	0.16	0.398
65	TM	-0.04	-0.15	0.06	0.417
66	RETN	-0.04	-0.15	0.06	0.433
67	PECAM-1	0.04	-0.07	0.15	0.461
68	FABP4	-0.05	-0.18	0.08	0.464
69	TNF-R2	-0.04	-0.15	0.07	0.487
70	IL-1ra	0.03	-0.08	0.15	0.559
71	CSF-1	0.03	-0.08	0.14	0.592
72	CCL3	0.03	-0.08	0.14	0.631
73	SRC	-0.03	-0.13	0.08	0.644
74	TF	-0.02	-0.13	0.08	0.669
75	ESM-1	-0.02	-0.13	0.09	0.684
76	MMP-10	0.02	-0.09	0.13	0.695
77	t-PA	0.02	-0.09	0.14	0.707
78	PSGL-1	0.02	-0.08	0.12	0.716
79	SELE	0.02	-0.09	0.13	0.730
80	CXCL6	-0.02	-0.12	0.09	0.734
81	SPON1	0.01	-0.10	0.12	0.878
82	VEGF-D	0.01	-0.10	0.12	0.884
83	FAS	0.006	-0.103	0.115	0.918
84	TNF-R1	-0.005	-0.116	0.105	0.926
85	CTSL1	0.005	-0.105	0.115	0.930
86	CASP-8	0.004	-0.103	0.111	0.940
87	hK11	0.004	-0.101	0.108	0.942

88	PlGF	0.001	-0.111	0.112	0.990

The beta coefficient, 95% confidence interval and p value were obtained from multiple linear regression performed separately for each protein.

Adjustments: age, sex, BMI, HGB, LDL, HDL, diabetes, smoking.

*: $p<5.68\times10^{-4}$.
