# Supplementary material for: The associations between red cell distribution width and plasma proteins in a general population
Source: Clin Proteomics. 2021 Mar 30;18:12. doi: 10.1186/s12014-021-09319-9 (PMC8008679; doi:10.1186/s12014-021-09319-9)
Supplement: Supplementary file 3 — Additional file 3: Table S3. The associations between red cell distribution width and plasma proteins in replication sample. [file 12014_2021_9319_MOESM3_ESM.pdf]

Table S3 The associations between red cell distribution width and plasma proteins in replication sample

			95% Confidence Interval		
	Plasma proteins	Beta coefficient	Lower bound	Upper bound	P value
1	GDF-15*	0.46	0.29	0.63	7.97×10^{-8}
2	SIRT2*	0.39	0.24	0.53	2.05×10^{-7}
3	ITGB1BP2*	0.35	0.21	0.49	1.80×10^{-6}
4	CHI3L1*	0.38	0.23	0.54	2.21×10^{-6}
5	MMP-7*	0.38	0.22	0.54	2.69×10^{-6}
6	SCF*	-0.36	-0.52	-0.21	5.59×10^{-6}
7	CD40-L*	0.34	0.19	0.49	8.48×10^{-6}
8	IL-8*	0.31	0.16	0.47	8.85×10^{-5}
9	HGF*	0.33	0.16	0.49	1.21×10^{-4}
10	U-PAR*	0.26	0.10	0.42	1.46×10^{-3}
11	MMP-3*	0.27	0.09	0.46	2.82×10^{-3}
12	PRL	0.11	-0.05	0.26	0.183
13	MB	-0.10	-0.26	0.06	0.232

The beta coefficient, 95% confidence interval and p value were obtained from multiple linear regression performed separately for each protein.
Adjustments: age, sex, BMI, HGB, LDL, HDL, diabetes, smoking.
*: $p<0.05$.
