# Supplementary material for: The associations between red cell distribution width and plasma proteins in a general population
Source: Clin Proteomics. 2021 Mar 30;18:12. doi: 10.1186/s12014-021-09319-9 (PMC8008679; doi:10.1186/s12014-021-09319-9)
Supplement: Supplementary file 4 — Additional file 4: Table S4. Pair-wise correlations between plasma proteins with significant relationships with RDW. [file 12014_2021_9319_MOESM4_ESM.pdf]

Table S4 Pair-wise correlations between plasma proteins with significant relationships with RDW

	GDF-15	SIRT2	ITGB1BP2	CHI3L1	MMP-7	SCF	CD40-L	IL-8	HGF	U-PAR	MMP-3
GDF-15	1	.24**	.24**	.51**	.49**	.12**	.28**	.49**	.66**	.66**	.39**
SIRT2		1	.89**	.15**	.36**	.14**	.87**	.46**	.38**	.27**	.20**
ITGB1BP2			1	.13**	.33**	.13**	.80**	.41**	.35**	.27**	.18**
CHI3L1				1	.34**	.05**	.19**	.36**	.48**	.45**	.32**
MMP-7					1	.17**	.36**	.46**	.49**	.47**	.24**
SCF						1	.14**	.18**	.22**	.25**	.21**
CD40-L							1	.47**	.41**	.29**	.20**
IL-8								1	.55**	.48**	.32**
HGF									1	.69**	.36**
U-PAR										1	.30**
MMP-3											1

Correlation coefficients were calculated between each two proteins using Pearson's correlation test.
**: $p<0.01$.
