# Supplementary material for: The associations between red cell distribution width and plasma proteins in a general population
Source: Clin Proteomics. 2021 Mar 30;18:12. doi: 10.1186/s12014-021-09319-9 (PMC8008679; doi:10.1186/s12014-021-09319-9)
Supplement: Supplementary file 5 — Additional file 5: Table S5. The associations between red cell distribution width and plasma proteins among never smokers. [file 12014_2021_9319_MOESM5_ESM.pdf]

Table S5 The associations between red cell distribution width and plasma proteins among never smokers

			95% Confidence Interval		
	Plasma proteins	Beta coefficient	Lower bound	Upper bound	P value
1	SCF*	-0.34	-0.48	-0.20	2.38×10^{-6}
2	ITGB1BP2*	0.25	0.12	0.38	1.26×10^{-4}
3	SIRT2*	0.25	0.12	0.37	2.13×10^{-4}
4	PRL*	0.22	0.09	0.35	7.85×10^{-4}
5	CHI3L1*	0.22	0.08	0.36	2.70×10^{-3}
6	MMP-7*	0.20	0.07	0.33	3.26×10^{-3}
7	GDF-15*	0.23	0.07	0.38	3.80×10^{-3}
8	CD40-L*	0.19	0.06	0.32	4.81×10^{-3}
9	IL-8*	0.17	0.04	0.29	9.90×10^{-3}
10	MMP-3*	0.21	0.05	0.38	0.011
11	HGF*	0.14	0.002	0.29	0.047
12	MB	-0.13	-0.28	0.006	0.061
13	U-PAR	0.08	-0.06	0.22	0.270

The beta coefficient, 95% confidence interval and p value were obtained from multiple linear regression performed separately for each protein.
Adjustments: age, sex, BMI, HGB, LDL, HDL, diabetes.
*: $p<0.05$.
