# Supplementary material for: The associations between red cell distribution width and plasma proteins in a general population
Source: Clin Proteomics. 2021 Mar 30;18:12. doi: 10.1186/s12014-021-09319-9 (PMC8008679; doi:10.1186/s12014-021-09319-9)
Supplement: Supplementary file 6 — Additional file 6: Figure S1. Red cell distribution width in relation to plasma proteins in discovery cohort. [file 12014_2021_9319_MOESM6_ESM.pdf]

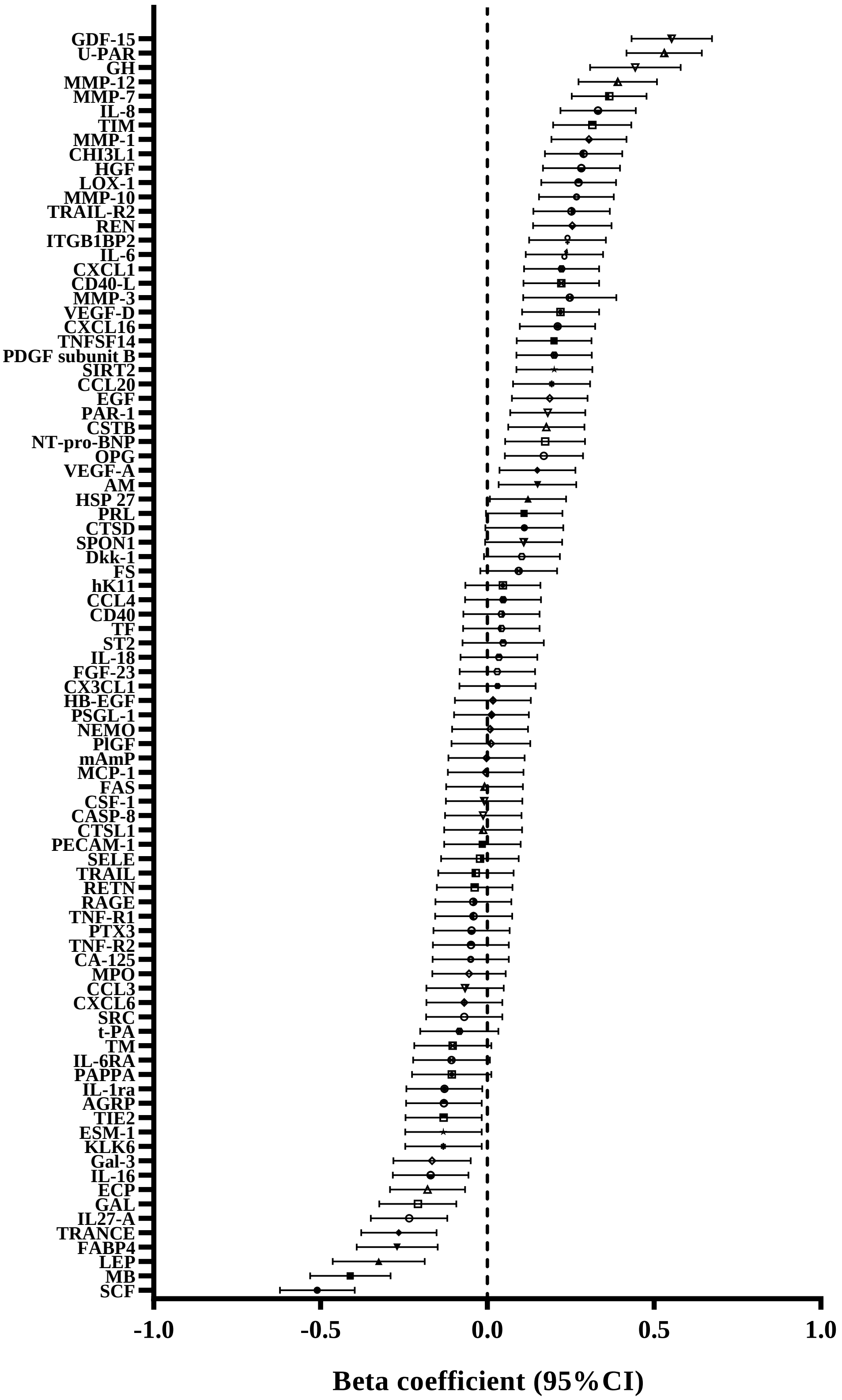

Figure S1: Red cell distribution width in relation to plasma proteins in discovery cohort

The beta coefficient and 95% confidence interval (CI) were obtained from multiple linear regression performed separately for each protein. Adjustments: age and sex. P $<5.68\times10^{-4}$ is significant.
