# Supplementary material for: The associations between red cell distribution width and plasma proteins in a general population
Source: Clin Proteomics. 2021 Mar 30;18:12. doi: 10.1186/s12014-021-09319-9 (PMC8008679; doi:10.1186/s12014-021-09319-9)
Supplement: Supplementary file 7 — Additional file 7: Figure S2. The associations between red cell distribution width and plasma proteinsamong never smokers. [file 12014_2021_9319_MOESM7_ESM.pdf]

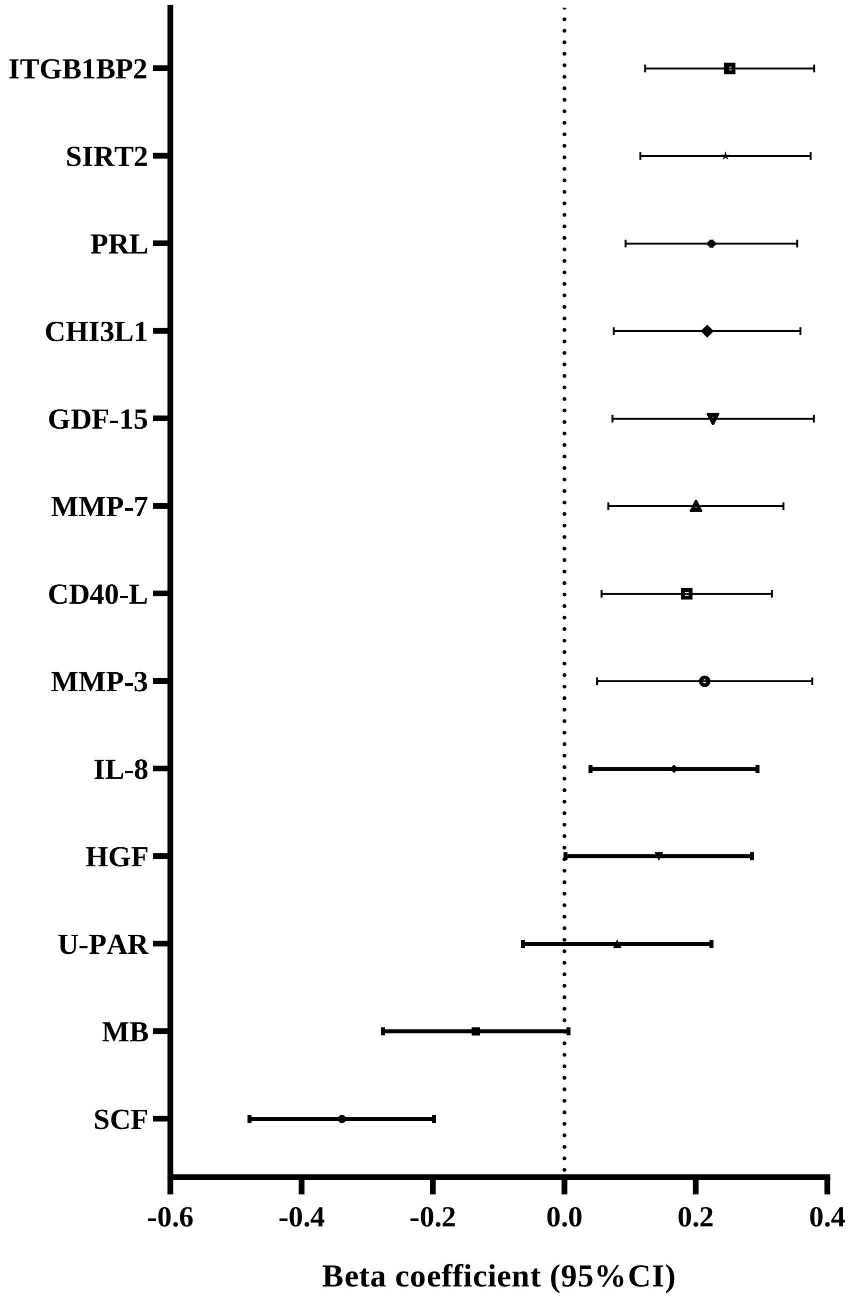

Figure S2: The associations between red cell distribution width and plasma proteins among never smokers

The beta coefficient and 95% confidence interval (CI) were obtained from multiple linear regression performed separately for each protein.
Adjustments: age, sex, BMI, HGB, LDL, HDL, diabetes.
P<0.05 is significant.
