# Supplementary figures and images for: The associations between red cell distribution width and plasma proteins in a general population
Source: Clin Proteomics. 2021 Mar 30;18:12. doi: 10.1186/s12014-021-09319-9 (PMC8008679; doi:10.1186/s12014-021-09319-9)

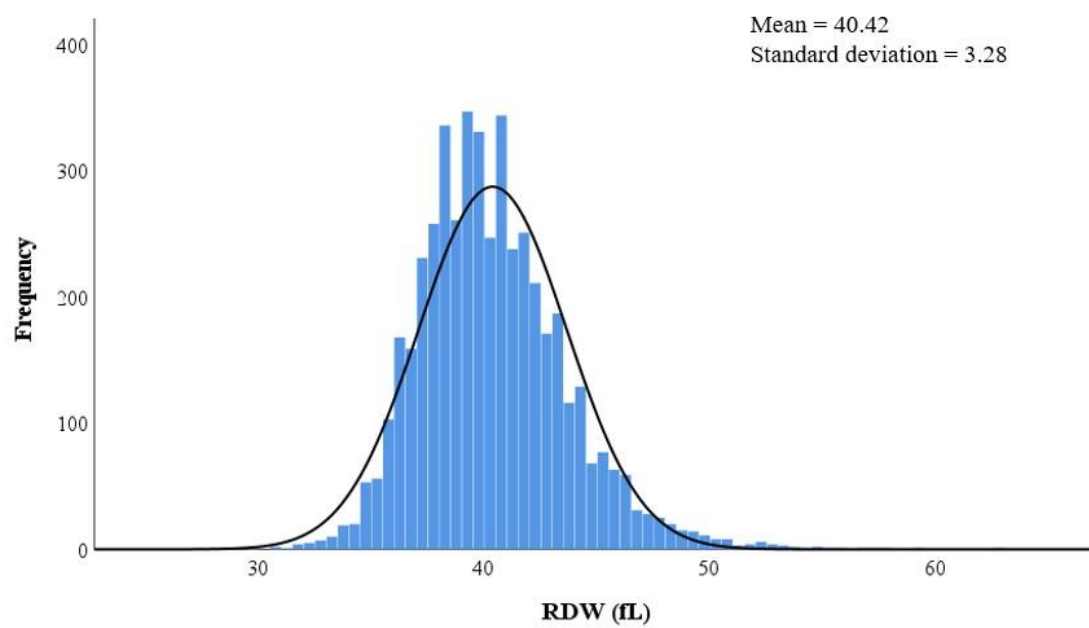

**Figure S3: Distribution plot for RDW**

RDW: red cell distribution width.

Supplement: Supplementary file 8 — Additional file 8: Figure S3. Distribution plot for RDW. [file 12014_2021_9319_MOESM8_ESM.pdf]
