# Supplementary material for: The associations between red cell distribution width and plasma proteins in a general population
Source: Clin Proteomics. 2021 Mar 30;18:12. doi: 10.1186/s12014-021-09319-9 (PMC8008679; doi:10.1186/s12014-021-09319-9)
Supplement: Supplementary file 9 — Additional file 9: Figure S4. The scatter plots for RDW and four validated proteins – (A) GDF-15, (B) SIRT2, (C) CHI3L1 and (D) SCF. [file 12014_2021_9319_MOESM9_ESM.pdf]

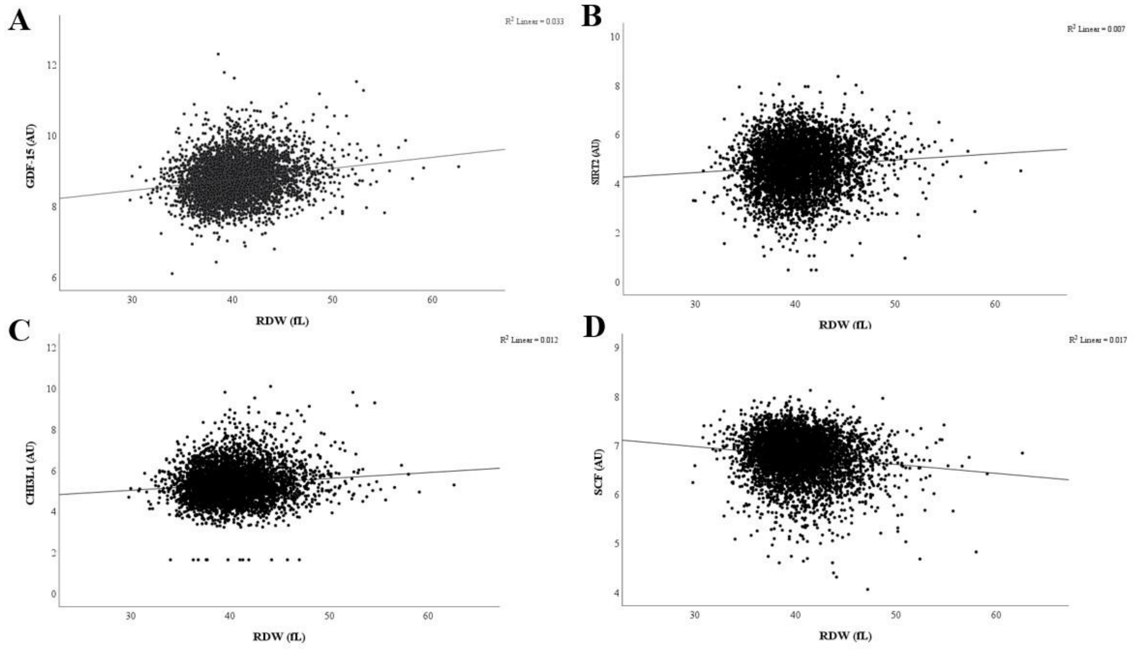

Figure S4: The scatter plots for RDW and four validated proteins – (A) GDF-15, (B) SIRT2, (C) CHI3L1 and (D) SCF.

GDF-15, growth differentiation factor-15; RDW: red cell distribution width; SIRT2: Sir-like protein 2; CHI3L1: Chitinase-3-like protein 1; SCF: stem cell factor; AU: arbitrary units, log2 scale.
